# Antimalarial Activity of Tri- and Tetra-Substituted Anilino Pyrazoles

**DOI:** 10.3390/molecules28041712

**Published:** 2023-02-10

**Authors:** Matteo Lusardi, Nicoletta Basilico, Chiara Rotolo, Silvia Parapini, Andrea Spallarossa

**Affiliations:** 1 Department of Pharmacy, University of Genova, Viale Benedetto XV, 3, 16132 Genova, Italy; 2 Dipartimento di Scienze Biomediche, Chirurgiche e Odontoiatriche, Università degli Studi di Milano, 20133 Milan, Italy; 3 Dipartimento di Scienze Biomediche per la Salute, Università degli Studi di Milano, 20133 Milan, Italy

**Keywords:** pyrazoles, regioselective synthesis, antiplasmodial activity, structure–activity relationships

## Abstract

Pyrazole core represents a privilege scaffold in medicinal chemistry; a number of pyrazole compounds are endowed with various pharmacological activities in different therapeutic areas including antimalarial treatment. Supported by this evidence, a series of 5-anilino-3-(hetero)arylpyrazoles were evaluated for their antiplasmodial activity in in vitro assays. The compounds were synthesized according to regioselective and versatile protocols that combine active methylene reagents, aryl isothiocyanates and (substituted)hydrazines. The considered derivatives **2** allowed the definition of consistent structure–activity relationships and compounds **2b**,**e**,**k**,**l** were identified as the most interesting derivatives of the series showing micromolar IC_50_ values against chloroquine-sensitive and chloroquine-resistant *Plasmodium* strains. Additionally, the most active anilino-pyrazoles did not show any cytotoxicity against tumor and normal cells and were predicted to have favorable drug-like and pharmacokinetic properties.

## 1. Introduction

Malaria is a life-threatening disease caused by protozoan parasites of the genus *Plasmodium* that are transmitted to humans through the bites of infected female *Anopheles* mosquitoes. In the human host, *Plasmodium* sporozoites reach peripheral circulation and migrate into the hepatocytes forming merozoites that are released into the bloodstream. Merozoites infect red blood cells and, upon proliferation, cause their lysis. A small proportion of blood stage parasites develop into sexual stages (i.e., gametocytes) that reach the dermis where they are taken up by another mosquito. Of the five Plasmodium species known to cause malaria in humans (namely, Plasmodium vivax, Plasmodium malariae, Plasmodium ovale, Plasmodium knowlesi and Plasmodium falciparum), Plasmodium falciparum is responsible for severe disease pathology and for the majority of deaths, especially in sub-Saharan Africa. Symptoms of malaria include fever and flu-like illness, headache, muscle aches, tiredness, nausea, vomiting and diarrhea. Malaria may cause anemia and jaundice and, if not promptly treated, the infection may cause kidney failure, seizures, mental confusion, coma and death. In 2021, 247 million malaria cases have been estimated with 619,000 deaths globally. Malaria is endemic in 84 countries, and 4 countries (namely, Nigeria, Democratic Republic of the Congo, Uganda, Mozambique) accounted for about 50% of all cases globally. The African Region is massively affected by malaria with an estimated 234 million cases (i.e., 95% of cases) in 2021 [1].

As reported by the WHO [2], the current therapeutic approach for malaria includes preventive (e.g., insecticides, use of chemoprophylaxis) and case management recommendations. In particular, pyrethroid-piperonyl butoxide (PBO) nets, pyrethroid-only long-lasting insecticides and indoor residual spraying proved to be effective interventions for vector control in malaria endemic regions. Additionally, malaria may be prevented by taking drugs that inhibit liver-stage development (causal prophylaxis; atovaquone + proguanil, primaquine) or drugs (e.g., chloroquine, proguanil, mefloquine) that kill asexual blood stages (suppressive prophylaxis). The use of malaria vaccine represents an effective strategy in malaria prevention. The RTS,S vaccine is the first and currently the only malaria vaccine to be recommended for use by WHO. RTS,S proved to significantly reduce (30% reduction) malaria deaths in children and was found to be safe and well tolerated [3]. Up to date, there is no malaria vaccine for adults who travel to a malaria-endemic area. Malaria case management is a crucial component of malaria control and elimination strategies. Early diagnosis and prompt, effective treatment of malaria based on artemisinin combination therapy (ACT) would prevent uncomplicated falciparum malaria to progress to severe forms of the disease. Following progressive reductions in malaria burden between 2000 and 2015, progress stalled as a consequence of the onset of drug-resistant *Plasmodium* species that undermine the efficacies of available antimalarial agents [4]. In this scenario, the development of novel antimalarial agents represents an urgent need [5] also considering the ambitious goal of the “Global technical strategy for malaria 2016–2030” for a world free of malaria by 2030 [6].

**Figure 1:**
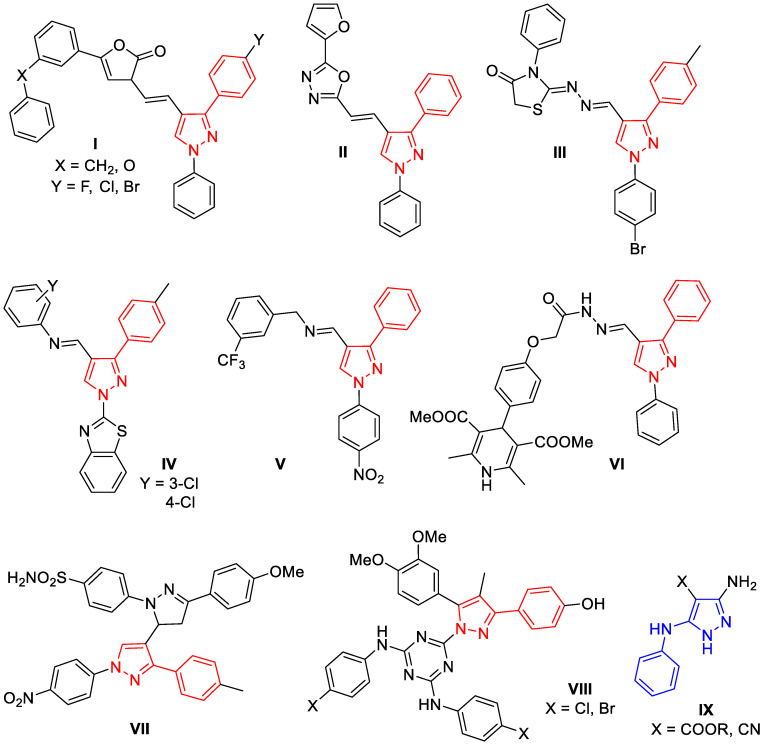
Tri- and tetra-substituted pyrazoles with antimalarial activity. The 3-phenylpyrazole and the anilino-pyrazole substructures are colored red and blue, respectively.

Pyrazole moiety represents a privileged scaffold in medicinal chemistry [7]. In fact, several pyrazole derivatives are currently used in clinic as anticancer (pyrazofurin), cytoprotective (crizotinib), anti-inflammatory (celecoxib and lonazolac), analgesic (difenamizole), anti-obesity (rimonabant), vasodilator (sildenafil) and antidepressant (fezolamide) drugs [8,9,10,11,12,13,14,15]. Moreover, the 3-phenyl pyrazole (compounds **I**–**VIII**, Figure 1) and the anilino-pyrazole (compounds **IX**, Figure 1) substructures are shared by several derivatives endowed with promising antimalarial pharmacological profiles. In particular, derivatives **I** [16] showed significative antimalarial properties in in vitro assays (i.e., schizont maturation inhibition and *Pf*LDH inhibition assay) resulting as active as chloroquine (CQ). Pyrazole **II** [17] was identified as a potent antimalarial agent effective also against CQ-resistant RKL 9 strain of *P. falciparum* and able to inhibit falcipain-2 enzyme (IC_50_ = 14 µM). Compound **III** resulted in five-fold more active than chloroquine against CQ resistant strains and docking simulations suggested its ability to inhibit *Pf*DHFR enzyme [18]. Pyrazole Shiff bases **IV** and **V** [19,20] were identified as antimalarial agents and compound **VI** showed nanomolar antiplasmodial IC_50_ value resulting more active than chloroquine, pyrimethamine and artimisinin [21]. Pyrazolylpyrazoline **VII** was endowed with antimalarial properties both in vitro and in vivo assays, thus pointing this derivative as a lead structure for the development of novel antimalarial agents [22]. Compounds **VIII** have been recently identified as promising antimalarial molecules whose activity can be further improved in future studies [23]. Anilino-pyrazoles **IX** (Figure 1) showed a good activity against *Plasmodium falciparum* in in vitro assays [24,25].

**Figure 2:**
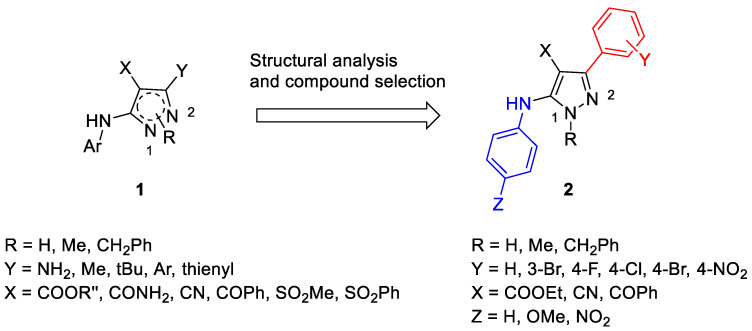
General structure of previously synthesized pyrazoles **1** and **2.** The 3-phenyl and the 5-anilino- substituents are colored red and blue, respectively.

Recently, we set up a regioselective one-pot procedure for the synthesis of highly functionalized anilino-pyrazoles **1** (Figure 2) [26,27]. The synthetic procedure involved the sequential condensation of active methylene reagents (AMRs), isothiocyanates and (substituted)hydrazines and proved to be versatile allowing the modification of the pyrazole nucleus with a wide variety of substituents. Tri- and tetra substituted pyrazoles **1** were tested against a panel of eight tumor cell lines (namely, breast cancer: MCF7, MDA-MB231, SK-Br3; melanoma: SKMEL-28; ovarian cancer: SKOV-3; liver cancer: Hep-G2; cervical cancer: HeLa; lung cancer: A549) and one normal human fibroblasts cell line (GM-6114). The prepared compounds proved to be non-cytotoxic against normal fibroblast cells, whereas selected members of the series emerged to be specifically active against SKMel-28 (melanoma) and HeLa (cervical cancer) cells [26,27]. Intriguingly, a subset of derivatives **1** (namely pyrazoles **2**, Figure 2) bear a 3-phenyl group and a 5-aniline moiety, i.e., both structural determinants characterizing the antimalarial pyrazoles **I**–**IX** (Figure 1).

**Scheme 1:**
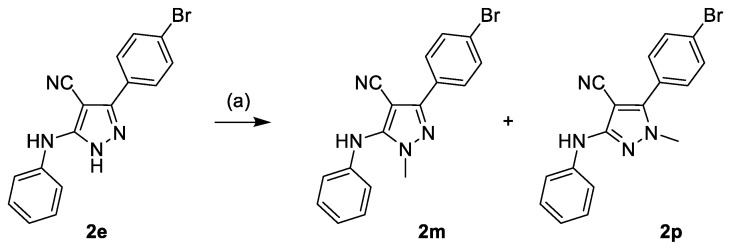
Synthesis of pyrazoles **2m** and **2p**. Reaction conditions: (a) DMF_dry_, K_2_CO_3_, CH_3_I, rt, 16 h.

The structural similarity of 5-anilino-3-(hetero)arylpyrazoles **2a**–**p** (Scheme 1) with previously reported antimalarial pyrazoles prompted us to evaluate their antiplasmodial activity against CQ-sensitive and CQ-resistant strains. To derive consistent structure–activity relationships (SARs), the effects on antimalarial activity of the following chemical variations have been considered: (i) substitution of the 3-phenyl ring with halogen or nitro groups; (ii) isosteric replacement of the 3-phenyl ring with a thienyl moiety; (iii) insertion at position 4 of the pyrazole scaffold of groups with different electronic and steric properties (i.e., CN, COOEt, COPh); (iv) para-substitution of the 5-aniline ring with electron withdrawing groups (i.e., OMe, NO_2_) and (v) methylation and benzylation of the pyrazole nitrogen atoms. 

## 2. Results and Discussion

### 2.1. Chemistry

Compounds **2a**–**l**,**n**,**o** were synthesized through an already published one-pot procedure that involved the sequential condensation of suitable AMRs (i.e., dibenzoylmethane, β-keto esters or β-keto nitriles), aryl isothiocyanates and (substituted)hydrazine [26]. Briefly, the reaction between AMR and isothiocyanate in the presence of sodium hydride led to the formation of *N,S*-thioketal intermediates that were *S*-methylated in situ. The addition of hydrazine, methylhydrazine or benzylhydrazine led to the displacement of the SMe group and the formation of the desired pyrazoles. In the adopted reaction conditions, the cyclization reaction emerged to be regioselective, leading to the formation of a single pyrazole isomer. In fact, after thiomethyl displacement, the hydrazinic amine group reacted selectively with the keto carbonyl group despite the electrophilic character of the X substituent (Table 1). Thus, the keto carbonyl group emerged to be more reactive than ester or cyano groups in the cyclization reaction [26,27].

The unreported pyrazoles **2m**,**p** were prepared by alkylation of compound **2e** following the previously reported procedure (Scheme 1) [27]. In particular, the methylation in anhydrous dimethylformamide of the pyrazole nitrogen with methyl iodide in the presence of potassium carbonate allowed the isolation of the two methyl-pyrazole isomers **2m** and **2p**. Despite their structural similarity, the two isomers showed different retention factors (R_f(**2m**)_ = 0.25; R_f(**2p**)_ = 0.13; eluent Et_2_O/ligroin 1:1 mixture) that allowed the isolation of the two compounds in 9% and 26% yields, respectively, after column chromatography.

According to previously adopted protocols [27], the structural assignment of the two pyrazole isomers were carried out by 2D-NMR spectroscopy (see Supplementary Materials). In particular, in the NOESY spectrum of **2m** the signal at {3.74;7.54} ppm indicates a spatial proximity among the protons of the *N*-methyl substituent and those of the anilino phenyl group. Additionally, the peak observed at {3.74, 146.40} ppm in the HMBC spectrum refers to the heteronuclear coupling between the *N*-methyl protons and the C-5 pyrazole carbon through a J^3^_C-H_ coupling constant thus confirming the unambiguous structural assignment of compound **2m**.

### 2.2. Antiplasmodial Activity

5-Anilino pyrazoles **2a**–**p** were screened for their antimalarial properties against the CQ-sensitive D10 and the CQ-resistant W2 *P. falciparum* strains. CQ was used as reference drug.

As reported in Table 1, compound **2a** showed a micromolar activity against both *Plasmodium* strains emerging slightly more active against the CQ-resistant strains. The substitution of 3-phenyl ring with a halogen atom (i.e., F, Cl or Br) at position 3 or 4 improves antimalarial properties of the compound (compare **2a** and **2b**–**e**). Interestingly, the bromo substitution maximized the gain in activity being derivatives **2b** and **2e** more active than their fluoro- or chloro-phenyl analogues **2c** and **2d** against both *Plasmodium* strains. However, the concomitant substitution of the 3-phenyl ring and the 5-anilino substructure as well as the isosteric replacement of the 3-phenyl group with a thienyl nucleus completely abolished activity (compare **2a** and **2f**; **2d** and **2g**,**h**). The nature of the substituent at position 4 of the pyrazole ring affect activity. Thus, the replacement of **2a** nitrile group with a benzoyl substituent caused the loss of activity (compare **2a** and **2i**) whereas the insertion of an ester functionality improved the antiplasmodial properties specially against the CQ-resistant W2 strain (compare **2a** and **2j**). Noteworthy, the introduction of a 4-nitrophenyl substituent at position 3 of the pyrazole ring led to a further improvement of activity, being derivative **2k** about 2-fold more potent than its unsubstituted analogue **2j** against D10 and W2 strains. Finally, the substitution of the pyrazole nitrogen atoms differently affected activity according to the nature of the *N*-substituent (Me or Bn), its position (*N*^1^ or *N*^2^) and the other groups decorating the pyrazole scaffold. Thus, the *N*^1^-methylation of 5-anilino-3-phenyl pyrazoles **2a** and **2i** considerably enhanced their antiplasmodial activity (compare **2a** and **2l**; **2i** and **2n**). Derivative **2l** represented the most active compound of the series with micromolar IC_50_ values against both *Plasmodium* strains. Conversely, the insertion of a *N*^1^-benzyl group did not significantly affect compound’s activity (compare **2a** and **2o**). Intriguingly, the *N*^1^- or *N*^2^-methylation of **2e** (a very close analogue of **2a**) proved to be detrimental for activity, being **2m** and **2p** less active that their trisubstituted analogue.

### 2.3. Pharmacokinetic Properties and Drug-Likeness Prediction

To evaluate the pharmaceutical relevance of the most active derivatives, the pharmacokinetics properties and the drug-likeness of compounds **2b**,**e**,**k**,**l** were calculated by SwissADME [28].

As detailed in Table 2, the low fraction of carbons in the sp^3^ hybridization would prevent oral bioavailability for all considered compounds **2** (optimal parameters: LogP range: −0.7 to +5; MW range: 150–500 g/mol; TPSA range: 20–130 Å^2^; Fraction Csp3 range: >0.25; number of rotatable bonds: 0–9). The compounds were predicted to have high gastrointestinal (GI) absorption and, with the exception of **2k**, a good blood–brain barrier (BBB) penetration. None of the derivatives would be a P-gp substrate. According to the calculations, all nitrile-substituted anilino-pyrazoles (namely, **2b**,**e** and **l**) would be able to inhibit cytochrome (CYP) isoforms 1A2, 2C9, 2D6 and 3A4, whereas the insertion of an ester functionality at position 4 (compound **2k**) would prevent the inhibition of CYP2D6 and CYP3A4 enzymes. The drug-like properties of derivatives **2** appear to be good, as no violations of the Lipinski rules were detected. Antimalarial pyrazoles **2** did not showed any pan assay interference compound (PAINS) alerts, whereas the presence of the nitro group in **2k** was spotted as problematic fragment according to the Brenk filters [29]. Noteworthily, this lead-likeness violation focused on the physicochemical boundaries defining a good lead (i.e., a small and hydrophilic compound suitable for optimization) and does not undermine the pharmaceutical potential of the compound series. A logP value higher than 3.5 (and for **2k** a molecular weight (MW) higher than 350 g/mol) has been estimated to be a limitation of the lead-likeness (i.e. a suitability for optimization) of derivatives **2b**,**e**,**k**,**l**, as implemented by Teague and co-workers [30].

## 3. Materials and Methods

### 3.1. Chemistry

Commercially available active methylene reagents, phenyl isothiocyanates, hydrazines and reagents (55% sodium hydride dispersion in mineral oil, iodomethane) were purchased by Alfa-Aesar and Sigma-Aldrich. DMF was reagent grade and was dried on molecular sieves (5Å 1/16" inch pellets). Unless otherwise stated, all commercial reagents were used without further purification. Organic solutions were dried over anhydrous sodium sulphate. A thin layer chromatography (TLC) system for routine monitoring the course of reactions and confirming the purity of analytical samples employed aluminum-backed silica gel plates (Merck DC-Alufolien Kieselgel 60 F254). Neat DCM or DCM/2% methanol mixture were used as a developing solvent and detection of spots was made by UV light and/or by iodine vapors. Melting points were determined on a Fisher-Johns apparatus and are uncorrected. ^1^H NMR and ^13^C NMR spectra were recorded on a Varian Gemini (Palo Alto, Palo Alto, CA, USA) or JEOL JNM-ECZR (Tokyo, Japan) instrument; chemical shifts were reported in δ (ppm) units relative to the internal reference tetramethylsilane, and the splitting patterns were described as follows: s (singlet), bs (broad singlet), d (doublet), t (triplet), q (quartet) and m (multiplet). The first order values reported for coupling constants J were given in Hz. Elemental analyses were performed by an EA1110 Analyzer, Fison Instruments (Milan, Italy). Compounds **2a**–**l**,**n**,**o** were prepared as previously reported [26,27].

#### Synthesis of Compounds **2m** and **2p**

A dry DMF solution (10 mL) of pyrazole **2e** (346 mg, 1 mmol) and anhydrous K_2_CO_3_ (168 mg, 1.2 mmol) was stirred at rt for 10 min. Methyl iodide (63 µL, 1 mmol) was added and the suspension was stirred at rt for 16 h. The sequential addition of water (10 mL) and 2N HCl (pH = 7) led to the isolation of a white solid which was collected by filtration. TLC analysis (eluent Et_2_O/ligroin 1:1 mixture) revealed two spots with Rf values of 0.25 (compound **2m**) and 0.13 (compound **2p**). The solid was dissolved in Et_2_O and the two compounds were separated by column chromatography (silica gel, eluent: Et_2_O/ligroin 1:1).

1-methyl-3-phenyl-5-(phenylamino)-1*H*-pyrazole-4-carbonitrile (**2m**). White solid. Mp 194–195 °C; Yield: 9%. ^1^H NMR (400 MHz, DMSO-*d*_6_): δ 3.74 (s, 3H, CH_3_N); 6.80–6.89 (m, 1H, arom. H); 7.19–7.29 (m, 2H, arom. H); 7.51–7.64 (m, 4H, arom. H); 7.79–7.87 (m, 2H, arom. H); 8.93 (bs, 1H, NH, exchangeable). ^13^C NMR (101 MHz, DMSO-*d*_6_) δ 37.68, 79.59, 114.22, 116.48, 119.99, 124.03, 125.88, 128.72, 131.22, 132.22, 141.95, 146.40, 151.99. Calcd for C_17_H_13_BrN_4_: C = 57.81; H = 3.71; N = 15.86. Found: C = 57.72; H = 3.68; N = 15.70.

5-(4-bromophenyl)-1-methyl-3-(phenylamino)-1*H*-pyrazole-4-carbonitrile (**2p**). White solid. Mp 202–204 °C; Yield: 26%. ^1^H NMR (400 MHz, DMSO-*d*_6_): δ 3.73 (s, 3H, CH_3_N); 6.90–6.99 (m, 3H, arom. H); 7.22–7.33 (m, 2H, arom. H); 7.67–7.84 (m, 4H, arom. H); 8.91 (bs, 1H, NH, exchangeable). ^13^C NMR (101 MHz, DMSO-*d*_6_) δ 36.06, 78.96, 114.38, 116.78, 121.37, 122.42, 127.81, 129.30, 130.35, 131.99, 141.54, 147.71, 148.85. Calcd for C_17_H_13_BrN_4_: C = 57.81; H = 3.71; N = 15.86. Found: C = 57.58; H = 3.51; N = 16.13.

### 3.2. Plasmodium Cultures and Compound Susceptibility Assay

Continuous in vitro *Plasmodium falciparum* cultures were carried out according to Trager and Jensen with slight modifications [32]. The CQ-susceptible strain D10 and the CQ-resistant strain W2 were maintained at 5% hematocrit (human type A-positive red blood cells) in Roswell Park Memorial Institute (RPMI) 1640 (EuroClone, Celbio) medium with the addition of 1% AlbuMax (Invitrogen, Milan, Italy), 0.01% hypoxanthine, 20 mM HEPES (at pH 7.4) and 2 mM glutamine. All the cultures were maintained at 37 °C in a low-oxygen standard atmosphere consisting of 1% O_2_, 5% CO_2_ and 94% N_2_. Compounds were dissolved in DMSO to a stock concentration of 10 mg/mL and then diluted with complete medium to achieve the required concentrations (final DMSO concentration <1%, which is non-toxic to the parasite). Derivatives were placed in 96-well flat-bottomed microplates in duplicate and seven 1:2 serial dilutions made directly in the plate in a volume of 100 µL. Asynchronous cultures with parasitemia of 1–1.5% (assessed through Giemsa stained blood smears) and 1% final hematocrit were aliquoted into the plates and incubated for 72 h at 37 °C in a final volume of 200 µL/well. RPMI was used as negative control whilst uninfected erythrocytes at 2% hematocrit were used as positive control. The antimalarial chloroquine (CQ) was tested against the parasite strains as a positive control of inhibition. Parasite growth was determined spectrophotometrically (OD_650_) by measuring the activity of the parasite lactate dehydrogenase (pLDH), according to a modified version of the method of Makler in control and drug-treated cultures [33]. The antimalarial activity is expressed as 50% inhibitory concentrations (IC_50_). The IC_50_ values were extrapolated from nonlinear regression analysis of the concentration–response curve, using the software Gen5 1.10 provided with the Synergy 4 (BioTek) reader. Each IC_50_ value is the mean of three independent experiments run in duplicates.

## 4. Conclusions

Highly functionalized 5-anilino-3-arylpyrazoles **2** were designed by substituting the pyrazole core with the two structural determinants characterizing antimalarial pyrazole (namely, 3-phenyl group and 5-anilino substructure). The designed molecular hybrids **2a**–**p** were tested for their antimalarial properties against CQ-sensitive and CQ-resistant *Plasmodium* strains. Derivativities **2b**,**e**,**k**,**l** showed interesting antimalarial properties being endowed with micromolar IC_50_ values against D10 and W2 *Plasmodium* strains. Interestingly, pyrazole **2l** emerged to be the most potent compound of the series and proved to be more effective against CQ-resistant strain (IC_50_ = 12.14 µM) than against CQ-sensitive strain (IC_50_ = 18.08 µM). Additionally, as previously reported [27], derivative **2l** did not exhibit significant cytotoxic activity against a panel of eight tumor cell lines and one normal human fibroblasts cell line at the concentration of 10 µM. Therefore, the significative antimalarial properties and the lack of cytotoxicity indicate **2l** as a promising lead structure. The SARs defined in the current study represent the basis for future lead-optimization focused on the identification of novel antimalarial agents particularly effective against CQ-resistant strains.

## Figures and Tables

**Table 1:** Antiplasmodial activity of 5-anilinopyrazoles **2**.

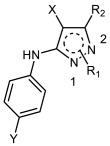						
					IC_50_ (µM) ^a^	
Cpd	R_1_	R_2_	X	Y	D10	W2
**2a**	H	C_6_H_5_	CN	H	65.60	52.96
**2b**	H	(3-Br)C_6_H_4_	CN	H	27.77	21.03
**2c**	H	(4-F)C_6_H_4_	CN	H	57.84	42.97
**2d**	H	(4-Cl)C_6_H_4_	CN	H	34.27	32.69
**2e**	H	(4-Br)C_6_H_4_	CN	H	19.75	19.87
**2f**	H	2-thienyl	CN	H	NA	NA
**2g**	H	(4-Cl)C_6_H_4_	CN	NO_2_	NA	NA
**2h**	H	(4-Cl)C_6_H_4_	CN	OMe	NA	NA
**2i**	H	C_6_H_5_	COPh	H	NA	NA
**2j**	H	C_6_H_5_	COOEt	H	54.48	38.25
**2k**	H	(4-NO_2_)C_6_H_5_	COOEt	H	25.67	18.74
**2l**	(1)-Me	C_6_H_5_	CN	H	18.08	12.14
**2m**	(1)-Me	(4-Br)C_6_H_4_	CN	H	NA	46.64
**2n**	(1)-Me	C_6_H_5_	COPh	H	36.29	30.43
**2o**	(1)-CH_2_C_6_H_5_	C_6_H_5_	CN	H	51.13	51.85
**2p**	(2)-Me	(4-Br)C_6_H_4_	CN	H	NA	44.67
**CQ**					0.03	0.47

^a^ Data are the mean of three experiments run in duplicate. NA = not active.

**Table 2:** Predicted pharmacokinetics and drug-like properties of anilino pyrazoles **2b**,**e**,**k**,**l.**

	2b	2e	2k	2l
*Physicochemical prop.*				
MW (g/mol)	339.19	339.19	352.34	274.32
Fraction Csp^3^	0.00	0.00	0.11	0.06
Rotatable bonds	3	3	7	3
H-bond acceptors	2	2	5	2
H-bond donors	2	2	2	1
TPSA ^a^ (Å^2^)	64.50	64.0	112.83	53.64
*Lipophilicity*				
LogP ^b^	4.71	4.71	4.35	4.04
*Water solubility*				
Solubility (mg/mL) ^c^	0.0017	0.0017	0.0058	0.0037
*Pharmacokinetics*				
GI absorption	High	High	High	High
BBB permeant	Yes	Yes	No	Yes
P-gp substrate	No	No	No	No
CYP1A2 inhibitor	Yes	Yes	Yes	Yes
CYP2C19 inhibitor	Yes	Yes	Yes	Yes
CYP2C9 inhibitor	Yes	Yes	Yes	Yes
CYP2D6 inhibitor	Yes	Yes	No	Yes
CYP3A4 inhibitor	Yes	Yes	No	Yes
*Druglikeness*				
Lipinski violations	0	0	0	0
*Medicinal chemistry*				
PAINS alerts	0	0	0	0
Brenk alerts	0	0	1	0
Leadlikeness violations	1	1	2	1

^a^ Topological polar surface area. ^b^ Predicted according to XLOGP3 program. ^c^ Values predicted by ESOL method [31].

## Data Availability

Data are contained within the article or supplementary material.

## Supplementary Materials

The following supporting information can be downloaded at: https://www.mdpi.com/article/10.3390/molecules28041712/s1, Figure S1. ^1^H-NMR (400 MHz, *d*_6_-DMSO) spectrum of compound **2m**. Figure S2. ^13^C-NMR (101 MHz, *d*_6_-DMSO) spectrum of compound **2m**. Figure S3. 2D NOESY (*d*_6_-DMSO) spectrum of compound **2m**. Figure S4. 2D HMBC (*d*_6_-DMSO) spectrum of compound **2m**. Figure S5. ^1^H-NMR (400 MHz, *d*_6_-DMSO) spectrum of compound **2p**. Figure S6. ^13^C-NMR (101 MHz, *d*_6_-DMSO) spectrum of compound **2p**.

## Sample Availability

Samples of compounds **2** are available from the authors.
