# Supplementary material for: Antimalarial Activity of Tri- and Tetra-Substituted Anilino Pyrazoles
Source: Molecules. 2023 Feb 10;28(4):1712. doi: 10.3390/molecules28041712 (PMC9964722; doi:10.3390/molecules28041712)
Supplement: Supplementary file 1 [file molecules-28-01712-s001.zip › molecules-2197390-supplementary.docx]

**Supporting Information**

**Antimalarial activity of tri- and tetra-substituted anilino pyrazoles**

Matteo Lusardi ^1^, Nicoletta Basilico ^2^, Chiara Rotolo ^1^, Silvia Parapini ^3^ and Andrea Spallarossa ^1,^*

1. Department of Pharmacy, University of Genova, viale Benedetto XV, 3, 16132, Genova, Italy; matteo.lusardi@edu.unige.it (M.L.); rotolo.chiara@libero.it (C.R.)
2. Dipartimento di Scienze Biomediche, Chirurgiche e Odontoiatriche, Università degli Studi di Milano, 20133 Milan, Italy; nicoletta.basilico@unimi.it (N.B.)
3. Dipartimento di Scienze Biomediche per la Salute, Università degli Studi di Milano, 20133 Milan, Italy; silvia.parapini@unimi.it (S.P.)

*Correspondence: andrea.spallarossa@unige.it (A.S.)

**Table of contents**

**Figure S1.** ^1^H-NMR (400 MHz, d_6_-DMSO) spectrum of compound **2m**

**Figure S2.** ^13^C-NMR (101 MHz, d_6_-DMSO) spectrum of compound **2m**

**Figure S3.** 2D NOESY (d_6_-DMSO) spectrum of compound **2m**

**Figure S4.** 2D HMBC (d_6_-DMSO) spectrum of compound **2m**

**Figure S5.** ^1^H-NMR (400 MHz, d_6_-DMSO) spectrum of compound **2p**

**Figure S6.** ^13^C-NMR (101 MHz, d_6_-DMSO) spectrum of compound **2p**

**
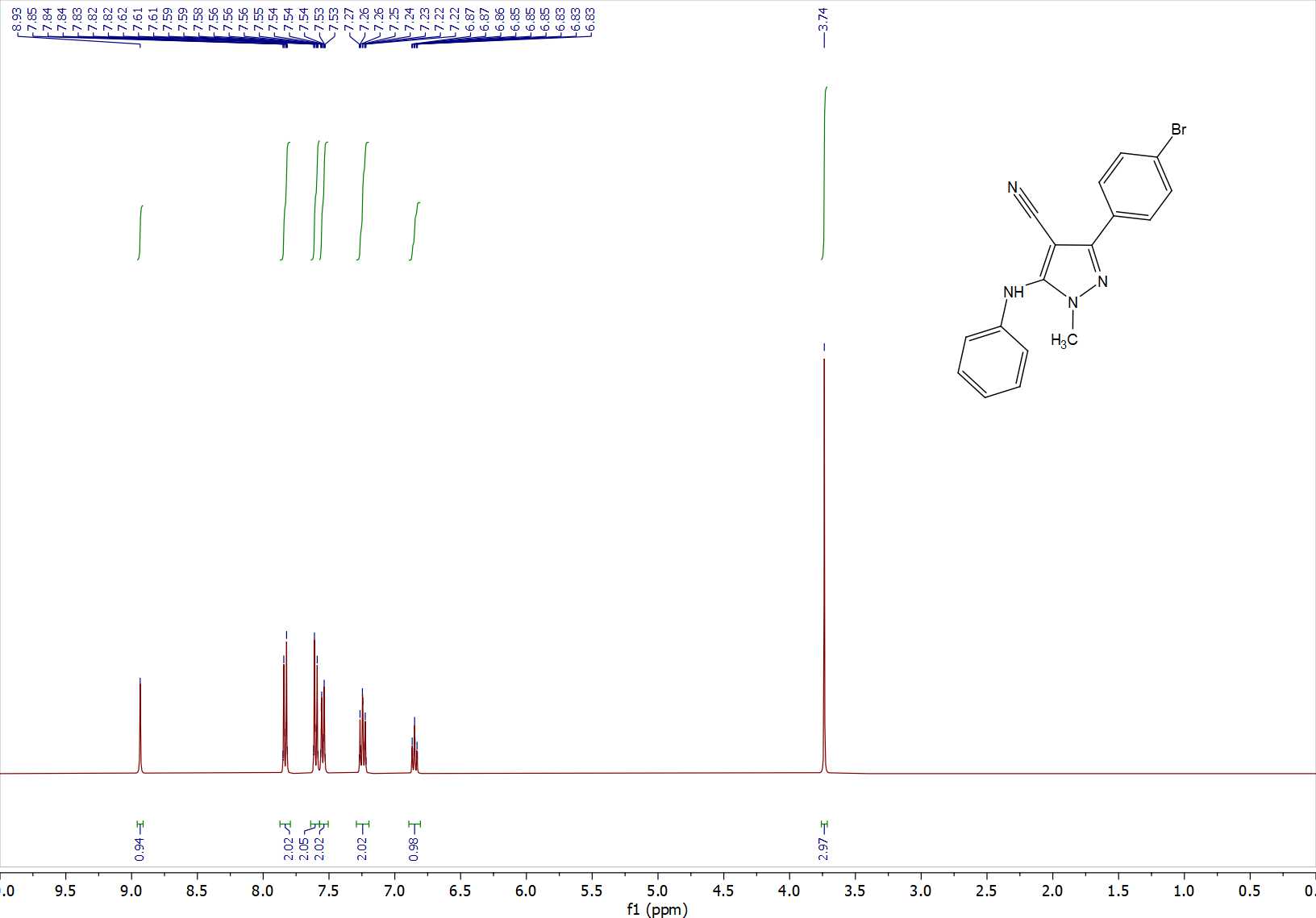
**

**Figure S1.** ^1^H-NMR (400 MHz, d_6_-DMSO) spectrum of compound **2m**

**
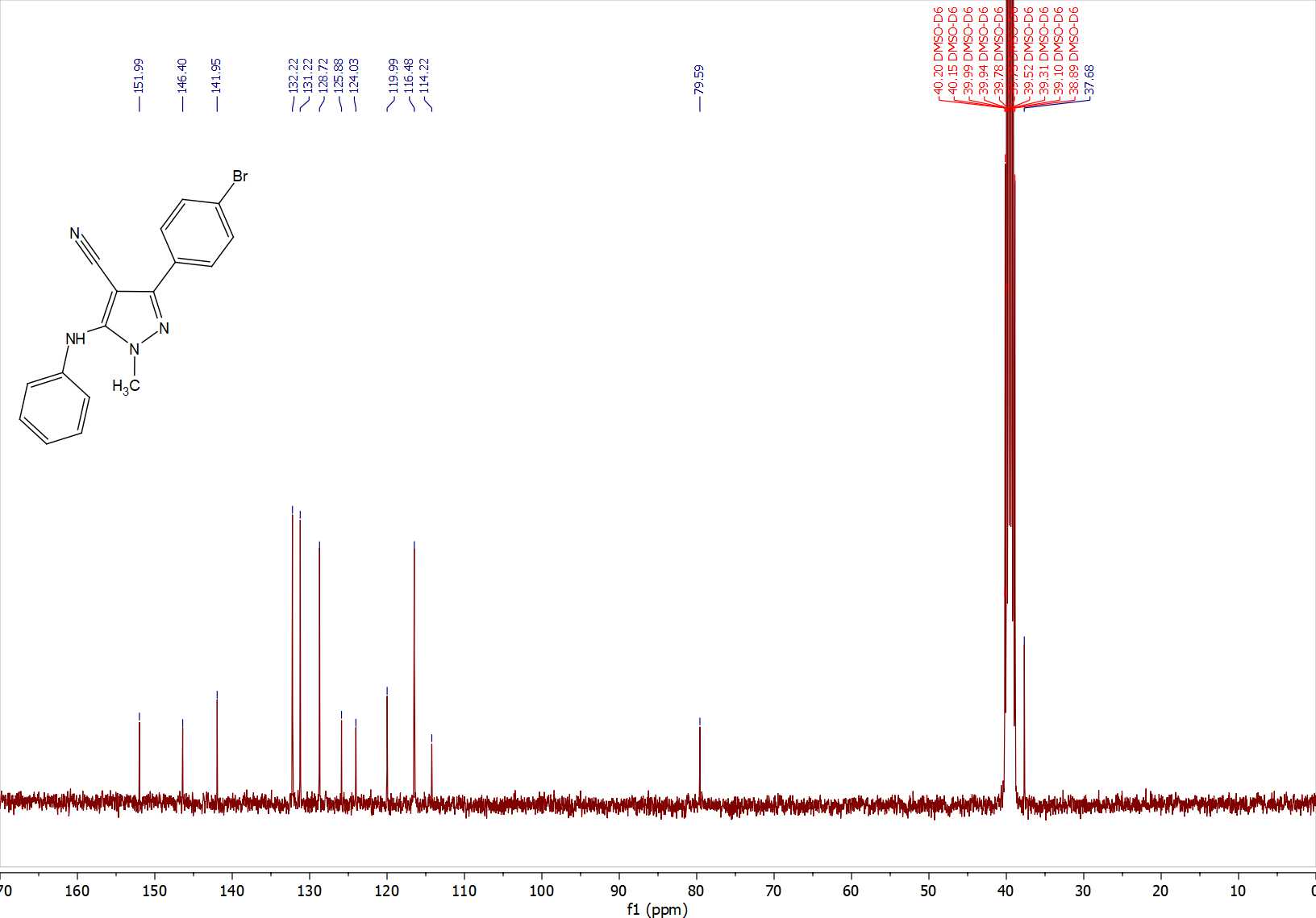
**

**Figure S2.** ^13^C-NMR (101 MHz, d_6_-DMSO) spectrum of compound **2m**

**
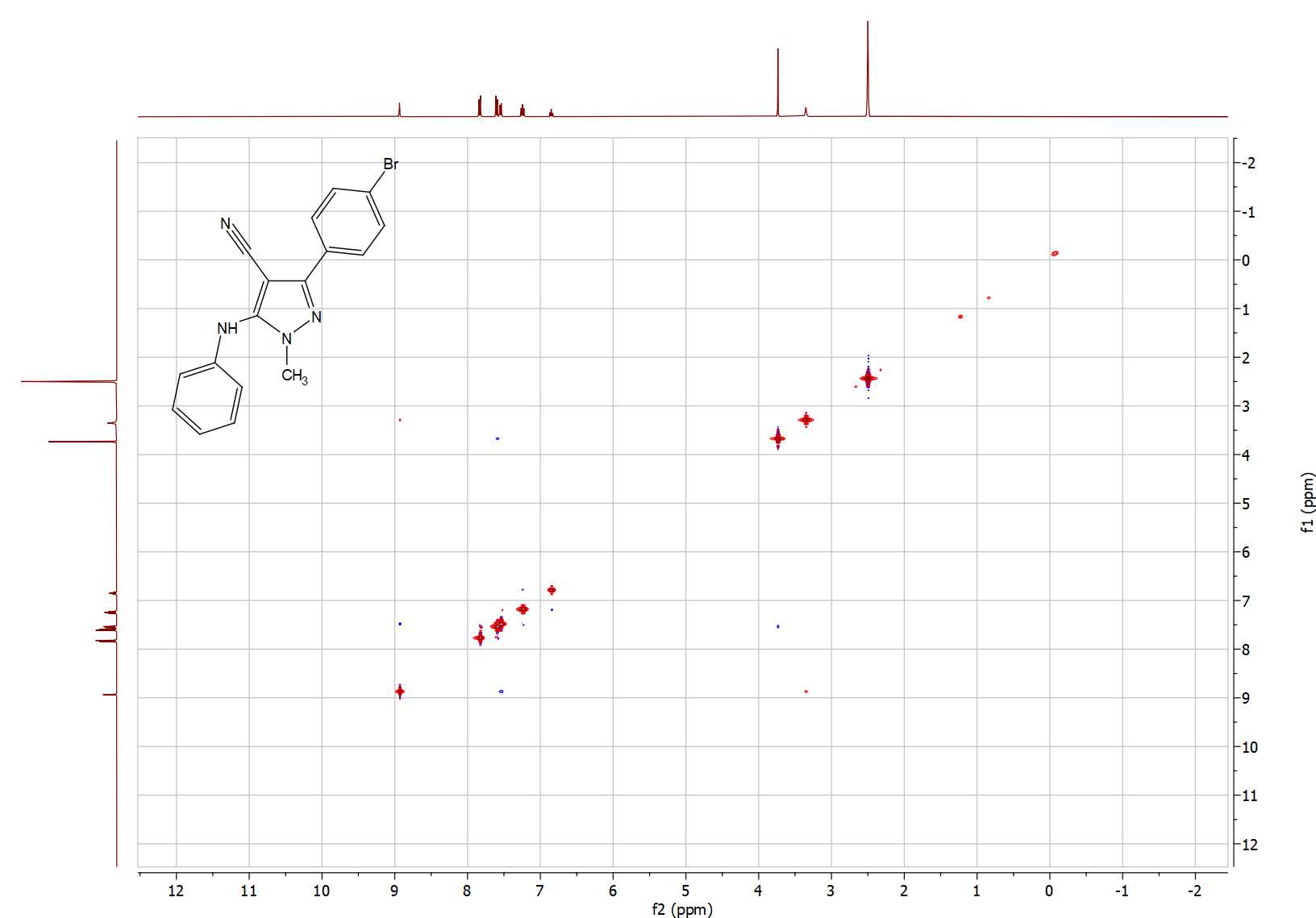
**

**Figure S3.** 2D NOESY (d_6_-DMSO) spectrum of compound **2m**

**
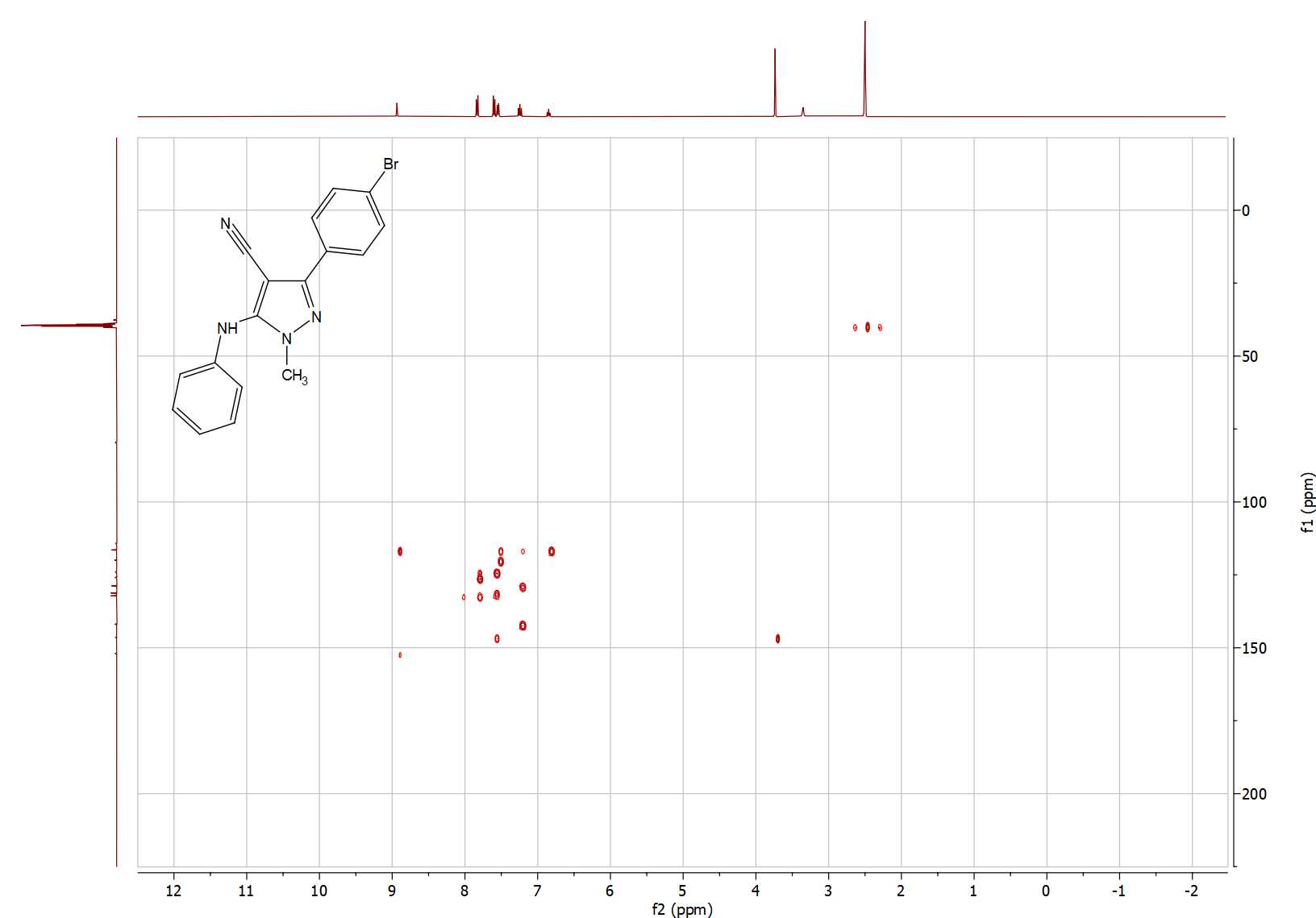
**

**Figure S4.** 2D HMBC (d_6_-DMSO) spectrum of compound **2m**

**
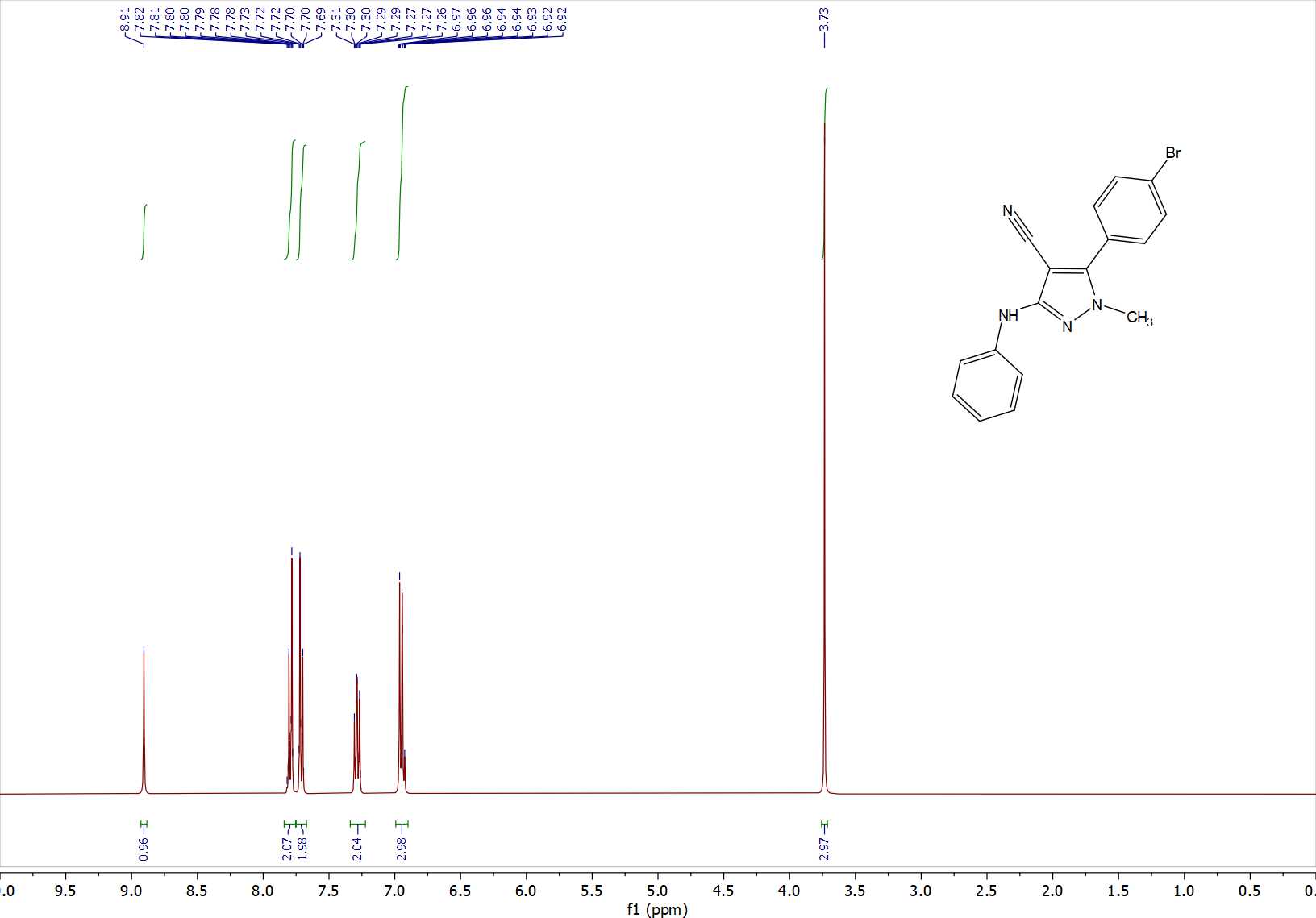
**

**Figure S5.** ^1^H-NMR (400 MHz, d_6_-DMSO) spectrum of compound **2p**

**
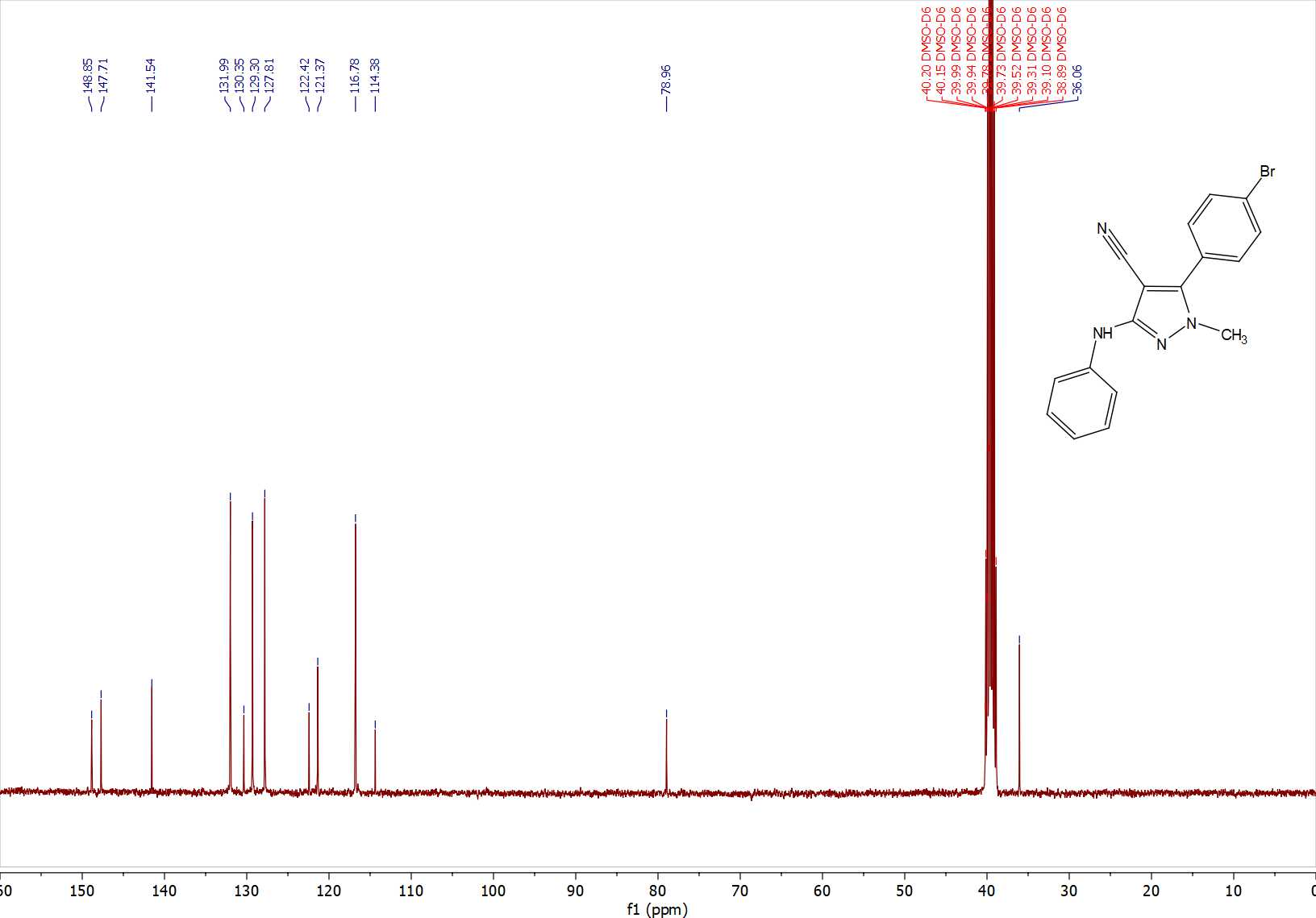
**

**Figure S6.** ^13^C-NMR (101 MHz, d_6_-DMSO) spectrum of compound **2p**
